# Single Dose of a VSV-Based Vaccine Rapidly Protects Macaques From Marburg Virus Disease

**DOI:** 10.3389/fimmu.2021.774026

**Published:** 2021-10-27

**Authors:** Andrea Marzi, Allen Jankeel, Andrea R. Menicucci, Julie Callison, Kyle L. O’Donnell, Friederike Feldmann, Amanda N. Pinski, Patrick W. Hanley, Ilhem Messaoudi

**Affiliations:** ^1^ Laboratory of Virology, Division of Intramural Research, National Institute of Allergy and Infectious Diseases, National Institutes of Health, Hamilton, MT, United States; ^2^ Department of Molecular Biology and Biochemistry, University of California, Irvine, Irvine, CA, United States; ^3^ Rocky Mountain Veterinary Branch, Division of Intramural Research, National Institute of Allergy and Infectious Diseases, National Institutes of Health, Hamilton, MT, United States

**Keywords:** Filovirus, MARV Angola, MVD, vesicular stomatitis virus, transcriptomics, time to immunity

## Abstract

Marburg virus (MARV) is a member of the filovirus family that causes hemorrhagic disease with high case fatality rates. MARV is on the priority list of the World Health Organization for countermeasure development highlighting its potential impact on global public health. We developed a vesicular stomatitis virus (VSV)-based vaccine expressing the MARV glycoprotein (VSV-MARV) and previously demonstrated uniform protection of nonhuman primates (NHPs) with a single dose. Here, we investigated the fast-acting potential of this vaccine by challenging NHPs with MARV 14, 7 or 3 days after a single dose vaccination with VSV-MARV. We found that 100% of the animals survived when vaccinated 7 or 14 days and 75% of the animal survived when vaccinated 3 days prior to lethal MARV challenge. Transcriptional analysis of whole blood samples indicated activation of B cells and antiviral defense after VSV-MARV vaccination. In the day -14 and -7 groups, limited transcriptional changes after challenge were observed with the exception of day 9 post-challenge in the day -7 group where we detected gene expression profiles indicative of a recall response. In the day -3 group, transcriptional analysis of samples from surviving NHPs revealed strong innate immune activation. In contrast, the animal that succumbed to disease in this group lacked signatures of antiviral immunity. In summary, our data demonstrate that the VSV-MARV is a fast-acting vaccine suitable for the use in emergency situations like disease outbreaks in Africa.

## Introduction

In 1967, a newly emerging pathogen caused hemorrhagic disease outbreaks with primary cases in Marburg, Germany (1) and Belgrade, Yugoslavia (2). Infected patients presented with fever, nausea, vomiting, conjunctivitis and later on petechiae as well as excessive bleeding suggesting clotting defects (3). In total, 32 people were infected, 7 of whom died (21.9% case fatality rate (CFR)) (1). Marburg virus (MARV) was identified as the causative agent and has since caused infrequent outbreaks in Africa with a CFR up to 90% (3). MARV is a member of the *Filoviridae*, has a 19 kb (-)single-stranded genome encoding 7 proteins, and forms filamentous particles that bud from the surface of infected cells (4). There is neither an approved treatment nor a vaccine against Marburg virus disease (MVD) and due to its high pathogenicity and effective human-to-human transmission MARV is classified as a select agent in the United States and the World Health Organization (WHO) added it to its list of priority pathogens (5).

Several vaccine approaches have been explored ranging from inactivated virus, subunit, and viral vector vaccines (6). One of the most promising vaccines is based on vesicular stomatitis virus (VSV) expressing the MARV glycoprotein (GP) as the viral antigen. A single dose of this vaccine is uniformly protective in nonhuman primates (NHPs) when administered 28 days prior to challenge (7, 8). It also demonstrated a great potential for use in a post-exposure scenario in NHPs (9). This vaccine is very similar to the VSV-based Ebola virus (EBOV) vaccine VSV-EBOV (also known as rVSV-ZEBOV) which has been approved by the US Food and Drug Administration (FDA) (10) and the European Medicines Agency (EMA) under the name “Ervebo” for human use to prevent Ebola virus disease (EVD) (11). A single dose of VSV-EBOV has been shown to protect NHPs and humans within 7-10 days from lethal disease (12, 13) and, more recently, it has been shown that a low dose of 10 PFU protects NHPs from disease (14). Because of certain similarities between MVD and EVD and their respective VSV-based vaccines, we wanted to explore the fast-acting potential of VSV-MARV in NHPs. We demonstrate that a single dose of VSV-MARV protects NHPs uniformly from lethal disease when administered up to 7 days before challenge. Notably, three out of four NHPs survived when the vaccine was administered 3 days prior to MARV infection with only one animal developing moderate disease. All control animals succumbed after developing classical signs of MVD as did one NHP in the day -3 vaccine group. In the day -3 group, whole blood (WB) transcriptional analysis revealed strong innate immune activation only in surviving NHPs. Collectively, these data demonstrate that the VSV-MARV is a fast-acting vaccine suitable for use in outbreak situations.

## Materials and Methods

### Ethics Statement

All the work involving infectious MARV was performed following standard operating procedures (SOPs) approved by the Rocky Mountain Laboratories (RML) Institutional Biosafety Committee (IBC) in the maximum containment laboratory at the RML, Division of Intramural Research, National Institute of Allergy and Infectious Diseases, National Institutes of Health. Animal work was performed in strict accordance with the recommendations described in the Guide for the Care and Use of Laboratory Animals of the National Institute of Health, the Office of Animal Welfare and the United States Department of Agriculture and was approved by the RML Animal Care and Use Committee (ACUC). Procedures were conducted by trained personnel under the supervision of veterinary staff on animals anesthetized with ketamine. All efforts were made to ameliorate animal welfare and minimize animal suffering in accordance with the Weatherall report on the use of nonhuman primates in research (https://royalsociety.org/policy/publications/2006/weatherall-report/). Animals were housed in adjoining individual primate cages that enabled social interactions, under controlled conditions of humidity, temperature, and light (12-h light:12-h dark cycles). Food and water were available *ad libitum*. Animals were monitored and fed commercial monkey chow, treats, and fruit at least twice a day by trained personnel. Environmental enrichment consisted of commercial toys, music, and video. Endpoint criteria based on clinical score parameters as specified and approved by the RML ACUC were used to determine when animals were humanely euthanized.

### Cells and Viruses

Vero E6 cells (mycoplasma negative) were grown at 37°C and 5% CO_2_ in Dulbecco’s modified Eagle’s medium (DMEM) (Sigma-Aldrich, St. Louis, MO) containing 10% fetal bovine serum (FBS) (Wisent Inc., St. Bruno, Canada), 2 mM L-glutamine, 50 U/mL penicillin, and 50 μg/mL streptomycin (all supplements from Thermo Fisher Scientific, Waltham, MA). VSV vaccines and challenge virus were propagated in Vero E6 cells using DMEM supplemented with 2% FBS, L-glutamine and penicillin/streptomycin. VSV-MARV expressing the MARV-Angola glycoprotein (GP) (8) and VSV-EBOV expressing the EBOV-Mayinga GP (15) were used for IM vaccination. MARV-Angola was obtained from the Public Health Agency of Canada (16) (GenBank accession number KY047763 (17)), propagated on Vero E6 cells, titered and stored in liquid nitrogen. All viruses were confirmed by sequencing and mycoplasma testing revealed no contaminants.

### Animal Cohorts and Study Design

Sixteen male and female cynomolgus macaques (*Macaca fascicularis*) 5-7 years of age and 4.0-6.7 kg in weight were used for this study. Three groups of cynomolgus macaques (n=4 per group) were vaccinated with a single intramuscular (IM) injection of 1x10^7^ plaque forming units (PFU) VSV-MARV at 14, 7 or 3 days before challenge. Control animals were IM-vaccinated with 1x10^7^ PFU VSV-EBOV (n=1 each day -14 and -7, n=2 day -3; n=4 total). Challenge was performed by IM injection of 1,000 PFU of MARV (confirmed by back-titration) as previously described (8). Clinical exams including a blood draw on anesthetized animals were conducted on days -14, -7, -3, 0, 3, 6, 9, 14, 21, 28, 35 and 42 post-challenge. The animals were observed at least twice daily for clinical signs of disease according to a RML ACUC-approved scoring sheet and humanely euthanized when they reached endpoint criteria. The study ended on day 42 post-challenge when all surviving animals were humanely euthanized.

### Hematology and Serum Chemistry

The total white blood cell, neutrophil, lymphocyte, and platelet counts were determined from EDTA blood with the IDEXX ProCyte DX analyzer (IDEXX Laboratories, Westbrook, ME). Serum biochemistry including aspartate aminotransferase (AST), alkaline phosphatase (ALP), glucose, creatinine, and total bilirubin was analyzed using the Piccolo Xpress Chemistry Analyzer and Piccolo General Chemistry 13 Panel discs (Abaxis, Union City, CA).

### Virus Loads

Viremia was determined from EDTA WB samples using Vero E6 cells (mycoplasma negative). To this end, cells were seeded in 48-well plates the day before titration. At the day of titration, blood samples were thawed, and 10-fold serial dilutions were prepared in DMEM without supplements. Media was removed from cells and triplicate wells were inoculated with each dilution. After one hour, DMEM supplemented with 2% FBS, penicillin/streptomycin and L-glutamine was added and cells were incubated at 37°C. Cells were monitored for cytopathic effect (CPE) and 50% tissue culture infectious dose (TCID_50_) was calculated for each sample employing the Reed and Muench method (18).

### Humoral Immune Responses

Post-challenge NHP sera were inactivated by gamma irradiation (5 MRad) (19) and removed from the maximum containment laboratory according to RML standard operating procedures (SOP) approved by the RML IBC. Antibody titers were determined using ELISA kits based on recombinant soluble MARV-Angola GPδTM (Alpha Diagnostics, San Antonio, TX). Serum samples were diluted 1:200 for assessment in the ELISA. The ELISA was performed, and titers were calculated as per manufacturer’s instructions.

### Serum Cytokine Levels

Serum samples were diluted 1:2 in serum matrix for analysis using the Milliplex Non-Human Primate Magnetic Bead Panel as per manufacturer’s instructions (Millipore, Burlington, MA). Concentrations for IL-1Ra, IL-8, IL-10, MCP-1, IFN-γ, IL-6, MIP-1α, IL-2 and IL-15 were determined for all samples. Values below the limit of detection of the assay were assigned the value of 1.

### cDNA Library Preparation for RNA Sequencing

RNA quality and concentration were assessed using an Agilent 2100 bioanalyzer. A total of 100 ng was used for library preparation. Total RNA samples were treated with 1 U of DNase I (New England BioLabs, Ipswich, MA) at 37°C for 10 min and cleaned with RNAClean XP beads (Beckman Coulter, Brea, CA). Ribosomal RNAs were depleted using rRNA removal beads (Illumina, San Diego, CA). The remaining RNA was fragmented at 94°C for 8 min to yield a median fragment size of 155 nucleotides (nt). Libraries were prepared using the TruSeq stranded total RNA library prep kit according to the instructions provided by the manufacturer (Illumina, San Diego, CA). Final library quality was assessed using an Agilent high-sensitivity DNA kit. Libraries with unique barcoded adaptors were pooled and sequenced in a single end read (1x100) on the Illumina NextSeq 500 platform.

### Bioinformatics Analysis

Data analysis was performed using the RNA-Seq workflow module of the systemPipeR package (20). RNA-Seq reads were demultiplexed, quality filtered, and trimmed using Trimmomatic with an average phred score cutoff of 30 and minimum length of 50 bp. Quality reports were generated with the FastQC function. Trimmed reads were mapped to the *Macaca fascicularis* reference genome (Macaca_fascicularis.Macaca_fascicularis_5.0.dna.toplevel.fa) using HISAT2 and the corresponding gene annotation (Macaca_fascicularis.Macaca_fascicularis_5.0.94.gtf) from Ensembl. Uniquely mapped reads were counted using summarizeOverlaps in strand-specific mode. Normalization and statistical validation of differentially expressed genes (DEGs) was performed using EdgeR package’s pair-wise function (21). 0 DPV data were used as the reference. DEGs were defined as protein coding genes with human homologues with at least a 2-fold change in expression, a multiple hypothesis Benjamini-Hochberg false discovery rate (FDR) corrected *P* value less than 0.05 and an average of at least 5 read per kilobase of transcript per million mapped reads (RPKM).

Temporal gene expression patterns and signatures that distinguish vaccine groups, survivors and non-surviving, and negative controls were analyzed using maSigPro, which is a two-way regression-based approach that finds a set of statistically significant DEGs for the entire time course (22). Only protein-coding genes with human homologs and an average of at least 5 read per kilobase of transcript per million mapped reads (RPKM) were included in this analysis.

### Functional Enrichment and Data Visualization

DEGs were first mapped to human homologs using BioMart (Ensemble Gene 94). Only protein-coding genes with human homologs were included for further analysis. The functional enrichment of DEGs was assessed using Metascape (23).

Heatmaps, Venn diagrams, bar graph and volcano were generated using R packages VennDiagram, dplyr, and ggplot2. Line graphs were generated using GraphPad Prism V8 (San Diego, CA).

### Statistical Analysis

Clinical data were examined for statistical significance using Prism version 9 (GraphPad, San Diego, CA). Survival curves were analyzed with Mantel-Cox test and values representing groups were analyzed by two-way ANOVA with Tukey’s multiple comparisons. Statistical analysis of the maSigPro data was carried out using Prism version 8 (GraphPad, San Diego, CA). Significance was determined using a one-way ANOVA with a Dunnett’s multiple-comparison test. Statistically significant differences are indicated as follows: p<0.0001 (****), p<0.001 (***), p<0.01 (**), and p<0.05 (*).

## Results

### VSV-MARV Vaccination Protects NHPs Within 7 Days From Lethal Disease

**Figure 1:**
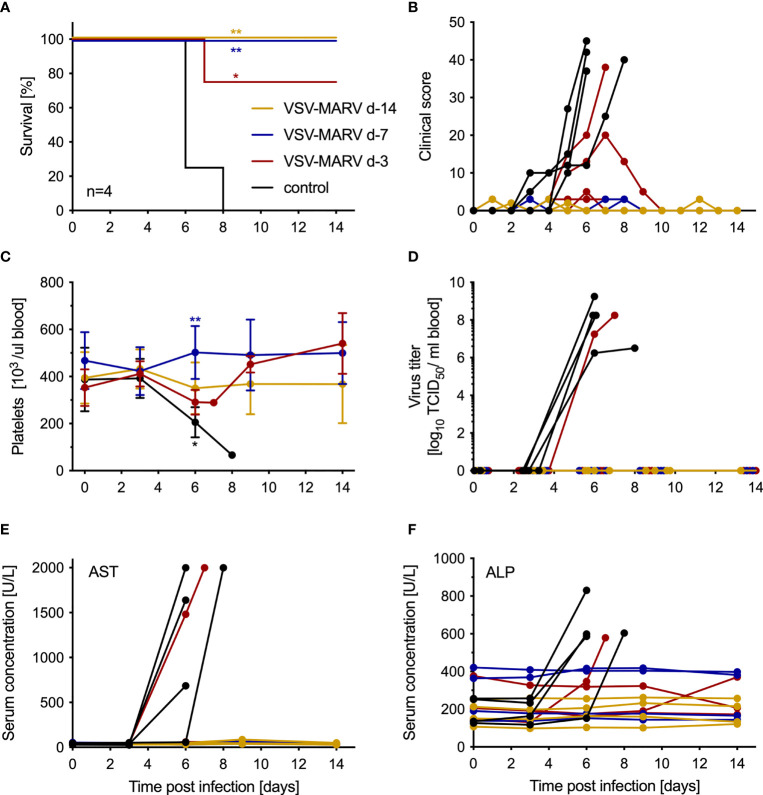
VSV-MARV vaccination protects NHPs within 7 days from lethal challenge. **(A)** Survival and **(B)** clinical score of vaccinated and challenged NHPs are shown. Statistical significance among survival rates was determined using Mantel-Cox test. Levels of **(C)** platelets, **(D)** MARV viremia, **(E)** aspartate aminotransferase (AST) and **(F)** alkaline phosphatase (ALP) in the blood or serum of MARV-infected NHPs. Mean and standard deviation (SD) are depicted in **(C)**. Statistical significance among groups was determined using two-way ANOVA with Tukey’s multiple comparisons. Statistical significance is indicated as **p < 0.01, and *p < 0.05.

The minimum time between vaccination and challenge for protection with a single IM dose of 1x 10^7^ PFU VSV-MARV was determined by vaccinating groups of 4 NHPs at 14, 7 and 3 days prior to challenge. The control group consisted of 4 VSV-EBOV-vaccinated NHPs that were vaccinated with a single IM dose of 1x 10^7^ PFU on day -14 (n=1), day -7 (n=1) and day -3 (n=2). On day 0, all NHPs were challenged with 1,000 PFU MARV by IM injection. The control animals developed signs of MVD and were humanely euthanized 6- and 8-days post-challenge (DPC) when they reached IACUC-approved endpoint criteria (**Figure 1A**). One NHP in the day -3 vaccine group developed clinical signs of MVD as demonstrated by the increase in clinical score (**Figure 1B**) and was euthanized 7 DPC (**Figure 1A**). Of the remaining three animals in the day -3 group, one developed moderate signs of MVD, two developed very mild signs of MVD, and all three recovered (**Figure 1B**). None of the NHPs in the day -14 or -7 vaccine groups developed signs of MVD (**Figure 1B**). Only the 5 NHPs that succumbed to disease developed hallmarks of MVD including thrombocytopenia (**Figure 1C**), high titer viremia (**Figure 1D**), and increased levels of AST (**Figure 1E**) and ALP (**Figure 1F**). Other parameters examined in WB and serum after challenge demonstrated changes to abnormal levels for cell populations and metabolites for the 5 NHPs that succumbed to MVD (**Figure S1**). Similarly, only these 5 NHPs developed cytokine levels indicative of the disease-associated cytokine storm (**Figure S2**).

### Surviving NHPs Developed MARV GP-Specific IgM and IgG Responses

**Figure 2:**
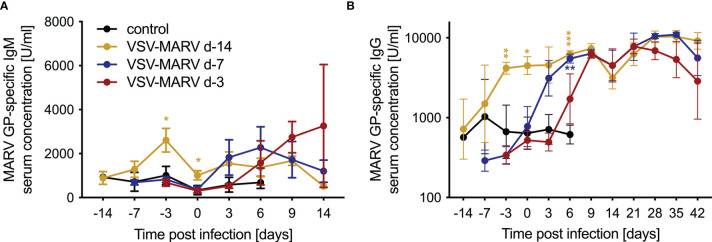
Humoral immune responses after MARV challenge. Concentrations of circulating MARV GP-specific **(A)** IgM and **(B)** IgG antibodies in the serum of vaccinated and challenged NHPs. Mean and SD are depicted. Statistical significance among groups was determined using two-way ANOVA with Tukey’s multiple comparisons. Statistical significance is indicated as ***p < 0.001, **p < 0.01, and *p < 0.05.

VSV-based vaccines have been shown to primarily confer protection by humoral responses (15, 24). Therefore, antigen-specific immune responses were determined in serum samples collected from all NHPs throughout the study. Levels of MARV GP-specific IgM peaked in the day -14 group 11 days post-vaccination (DPV; -3 DPC) before dropping by DPC 0 (**Figure 2A**). MARV GP-specific IgM was detected in the day -7 group 3 DPC increasing slightly 6 DPC dropping 9 and 14 DPC (**Figure 2A**). In the day -3 group MARV-GP specific IgM was detected 6 DPC increasing slightly 9 and 14 DPC (**Figure 2A**). Levels of MARV GP-specific IgG were detected 11 DPV (day -14 group), 3 DPC (day -7 group) and 9 DPC (day -3 group) (**Figure 2B**) coinciding with peak levels of MARV GP-specific IgM (**Figure 2A**). For the day -14 vaccination group the MARV challenge served as a boost as demonstrated by the increase in MARV GP-specific IgG 6 and 9 DPC (**Figure 2B**).

### VSV-MARV Vaccination Induces Transcriptional Changes Indicative of Antiviral Defense and B Cell Activation

**Figure 3:**
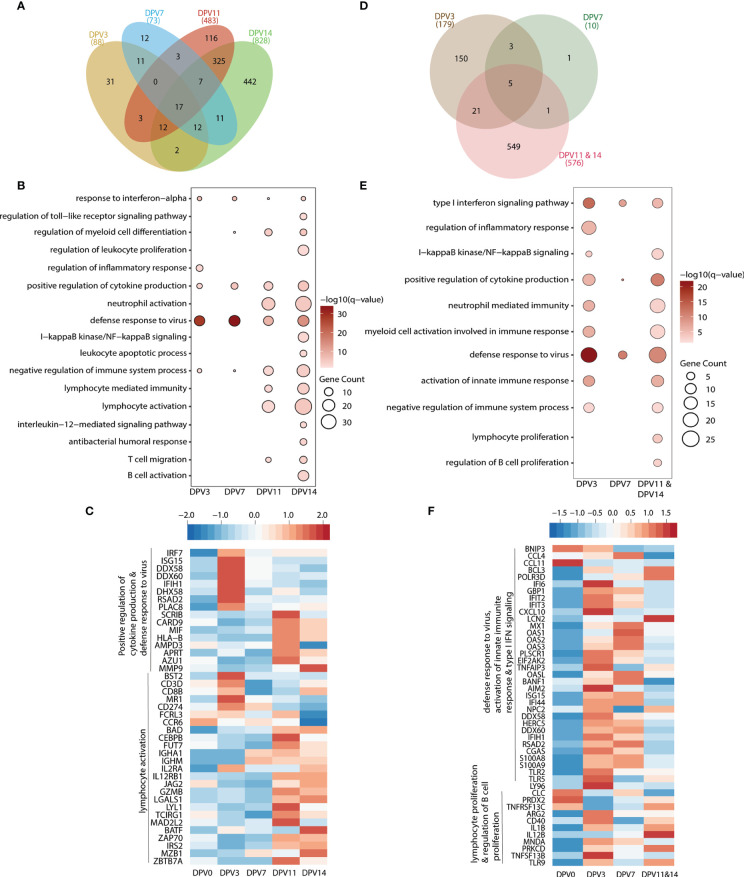
VSV-MARV vaccination induces transcriptional changes indicative of antiviral defense and B cell activation. **(A, D)** Venn diagram of differentially expressed genes (DEGs) (DEGs, defined as protein coding genes with human homologs with ≥ 2 fold change and false discovery rate (FDR) corrected p-value ≤ 0.05) detected in **(A)** VSV-MARV at 3 (n=8), 7 (n=8), 11 (n=4), and 14 (n=4) DPV relative to 0 DPV or VSV-EBOV at 3 (n=3), 7 (n=2), and 11/14 (n=2) DPV relative to 0 DPV. **(B, E)** Functional enrichment of DEGs detected 3, 7, 11, and 14 DPV; each bubble represents a Gene Ontology term; the size of each bubble represents number of DEGs enriching to that GO terms while color intensity represents the statistical significance (shown as –log_10_ of the q-corrected p-value). **(C, F)** Heatmap of DEGs enriched to **(C)** “positive regulation of cytokine production”, “defense response to virus” and “lymphocyte activation”; **(F)** “defense response to virus”, “activation of innate immune response”, “type I interferon signaling pathway”, “lymphocyte proliferation”, and “regulation of B cell proliferation.” Each column represents the median normalized transcript counts (RPKM) for each gene at each time point. The range of colors is based on scaled and centered RPKM values of the entire set of genes, with red indicating highly expressed genes and blue indicating lowly expressed genes.

Longitudinal transcriptional changes in WB in response to VSV-MARV and VSV-EBOV vaccination were assessed using RNA sequencing (RNA-Seq). To that end, transcriptomes of all available WB samples collected on 3, 7, 11, and 14 DPV were compared to those observed 0 DPV. Gene expression changes were evident as early as 3 DPV with VSV-MARV with 88, 73, 483 and 828 DEGs detected 3, 7, 11, and 14 DPV, respectively (**Figure 3A**). The largest number of DEGs was detected 14 DPV (**Figure 3A**). DEGs enriching to gene ontology (GP) terms associated with innate immunity to viral infection such as “defense response to virus” and “response to interferon alpha” were detected 3 DPV and remained differentially expressed throughout 14 DPV (**Figure 3B**). Notable DEGs in this group include interferon stimulated genes (ISG; *ISG15*, *BST2*, *IRF7*, *IFIH1*) as well as genes that play a role in antigen presentation (*HLA*-*B*), cytokine production (*MIF*) and antiviral response (*RSAD2, CARD9, DDX58, DDX60*, and *DHX58*) (**Figure 3C**). DEGs important for lymphocyte activation and adaptive immunity (e.g., GO terms “lymphocyte activation” and “lymphocyte mediated immunity”) were detected 11 DPV and included genes important for T cell activation and effector functions (*CD3D, CD8B*, *IL2RA*, *IL2RB1*, *GZMB* and *CD274*). DEGs important for B cell activation and antibody secretion (*MZB1*, *BATF*, *IGHM*, *IGHA1*) were upregulated 14 DPV (**Figures 3B, C**).

Samples collected 3, 7, and 11/14 DPV following VSV-EBOV vaccination were also compared to 0 DPV. Samples collected 11 and 14 DPV were combined because of the small number of animals. We detected 179, 10, and 576 DEGs at 3, 7, and 11/14 DPV, respectively (**Figure 3D**). As described for VSV-MARV above, DEGs important for innate immune response to virus were detected as early as 3 DPV and enriched to GO terms “type I interferon signaling pathway” and “defense response to virus” (**Figure 3E**). These DEGs included genes important for anti-viral defense (*OAS1*, *OAS2*, *OASL, MX1*); interferon stimulated genes (*IFIT2, IFIT3, IFI44, ISG15*); and inflammation (*S100A8, S100A9, CXCL10*) (**Figure 1F**). DEGs associated with lymphocyte activation and B cell proliferation were detected 11/14 DPV (GO terms “regulation of B cell proliferation” and “lymphocyte proliferation”) (**Figure 3E**) including *ARG2*, *CD40*, *IL12B*, *TNFSF13B* and *TNFRSF13C* (**Figure 3F**).

### Animals Vaccinated as Early as 7 Days Prior to Challenge Generate a Recall Adaptive Immune Response and Show Limited Gene Expression Changes

**Figure 4:**
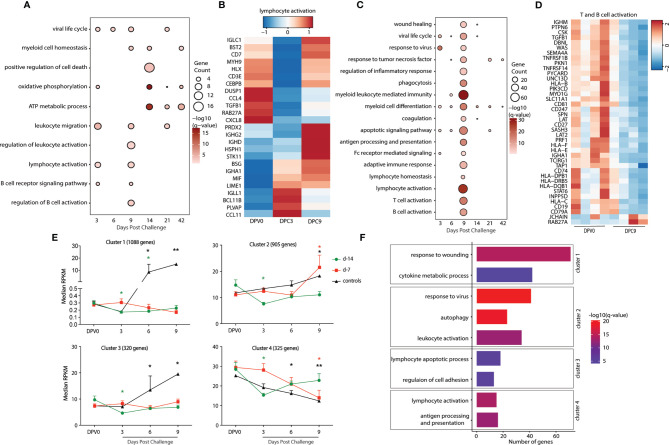
Animals vaccinated as early as 7 days prior to challenge generate a recall adaptive immune response. **(A, C)** Functional enrichment of DEGs post VSV-MARV challenge **(A)** day -14 group and **(C)** day -7 group; each bubble represents a Gene Ontology (GO) term and the size of each bubble represents number of DEGs enriching to that GO term while color intensity represents the statistical significance (shown as –log_10_ of the q-corrected p-value). **(B, D)** Heatmap of DEGs post VSV-MARV challenge enriching to (20) “lymphocyte activation”, “T cell activation”, and “B cell activation.” Each column represents the median normalized transcript counts (RPKM) for each gene at each time point. The range of colors is based on scaled and centered RPKM values of the entire set of genes, with red indicating highly expressed genes and blue indicating lowly expressed genes. **(E)** maSigPro analysis identified four distinct and significant temporal expression clusters of genes. The median RPKM of each cluster over time is plotted. Statistical significance among time points was determined using one-way ANOVA with Dunnett’s multiple-comparison test relative to 0 DPV. Statistical significance is indicated as **p < 0.01, and *p < 0.05. **(F)** Functional enrichment of the four clusters presented in **(E)**. Horizontal bars represent the number of genes mapping to each GO term with color intensity representing the negative log of the FDR-adjusted p-value -log(q-value).

Next, we examined gene expression changes post MARV challenge in the day -14 and -7 groups. All comparisons were made relative to 0 DPV in a pairwise manner in each group. Limited transcriptional changes were detected with the exception of 9 DPC in the day -7 group (**Table 1**). DEGs detected in the day -14 group at 3 and 6 DPC mapped to GO terms associated with viral life cycle, leukocyte migration, and lymphocyte activation (**Figure 4A**). DEGs important for lymphocyte activation include genes involved in T cell activation (*CD3E*, decreased 9 DPC), and B cell activation and antibody secretion (*IGLL1*, *IGHG2*, *IGHD*, and *IGHA1*; increased 9 DPC) (**Figure 4B**). DEGs detected 14-42 DPC played a role in “oxidative phosphorylation” and “ATP metabolic process” including (*ATP5F1D*, *ATP5F1E*, *COX2*, *NDUFA2*) (**Figure 4A**).

**Table 1:** Comparison of DEGs relative to 0 DPV.

Pairwise comparison	No. upregulated DEGs	No. downregulated DEGs
**Challenge 14 DPV**		
3 DPC *vs.* 0 DPV	58	7
6 DPC *vs.* 0 DPV	40	4
9 DPC *vs.* 0 DPV	71	2
14 DPC *vs.* 0 DPV	81	15
21 DPC *vs.* 0 DPV	47	4
42 DPC *vs.* 0 DPV	94	55
**Challenge 7 DPV**		
3 DPC *vs.* 0 DPV	45	19
6 DPC *vs.* 0 DPV	10	23
9 DPC *vs.* 0 DPV	101	604
14 DPC *vs.* 0 DPV	18	12
21 DPC *vs.* 0 DPV	13	23
42 DPC *vs.* 0 DPV	21	80
**Challenge 3 DPV**		
3 DPC *vs.* 0 DPV	42	2
6 DPC *vs.* 0 DPV	197	588
9 DPC *vs.* 0 DPV	47	41
14 DPC *vs.* 0 DPV	25	5
21 DPC *vs.* 0 DPV	35	15
42 DPC *vs.* 0 DPV	127	118

DEGs are defined as those with at least 2-fold change in expression relative

to 0 DPV with an FDR-corrected p value <0.05 and an average RPKM of 5.

The DEGs listed are protein-coding and human homolog genes.

DEG, differentially expressed genes; DPV, days post-vaccination; No., number; DPC, days post-challenge.

As described above, transcriptional changes detected in the day -7 group following MARV challenge enriched to GO processes associated with viral life cycle, myeloid cell differentiation, and apoptotic signaling pathway (**Figure 4C**). DEG that enriched to GO term “viral life cycle” included genes that play a role in viral budding (*VPS28*, *VPS37B*, *GRK2*), and Interferon-stimulated genes and chemoattractant (*ISG15*, *ISG20*, *CXCL8*, *CCL5*). DEGs important for adaptive immunity are detected 9 DPC with DEGs enriching to “T cell activation”, and “B cell activation” including DEGs that played a role in antigen presentation as well as T and B cell activation (*CD74*, *HLA-DRB5*, *HLA-C, CD247*, *CD19*, *CD79A*, *IGHA1*, and *CD27*) (**Figure 4D**). Interestingly, DEGs enriching to GO terms associated with lymphocyte activation were down regulated 9 DPC (**Figure 4D**). Finally, DEGs detected 14-42 DPC played a role in “myeloid cell differentiation” including *ITGA2B*, *SLC4A1*, *STAT5B*, *H3C11*, and *EPB42* (**Figure 4C**).

To better understand the temporal patterns of gene expression changes and signatures that distinguish the day -7 group, day -14 group, and negative controls, we used maSigPro (22). This approach provides a set of statistically significant DEGs for the entire time course rather than at each time point. This analysis identified 4 clusters with temporal expression changes that were significantly different between the groups (**Figure 4E**). Expression of genes in cluster 1 (1088 genes) either decreased 3 and 6 DPC (day -14 group) or remained unchanged (day-7 group) in vaccinated animals but increased in the controls 6 and 9 DPC (**Figure 4E**). Functional enrichment indicated that these genes were associated with inflammatory processes such as “response to wounding” (e.g., *F2*, *SERPINC1*, *APOH*, *ARRB1*) and “cytokine metabolic process” (e.g., *GDNF*, *IL34*, *VEGFC*, *CXCL6*) (**Figures 4F**, **S3A**). Genes in cluster 3 (320) had similar expression patterns as genes in cluster 1 (unchanged in day -7 group, decreased at 3 DPC in the day -14 group, significantly increased in controls). These genes enriched to “lymphocyte apoptotic process” (*FAS*, *BTK*, *CRKL*, *LGALS9*, *LGALS14*, *STAT3*) and “regulation of cell adhesion” (*ITGA5*, *PAK1*, *ADAM19*) (**Figures 4F** and **S3A**). Expression of genes in cluster 2 (905) increased 9 DPC in both day -7 group and controls but decreased in in day -14 group 3 DPC. These genes mapped to GO terms “response to virus” (e.g., *DHX9*, *IRF7*, *MX2*, *OAS2*, *STAT2*, *TLR8*), “autophagy” (e.g., *MAPK8*, *CASP1*, *ANXA7*, *VPS13A*, *VPS13C*, *VPS35*), and “leukocyte activation” (e.g., *CD44*, *CD53 CD59*, HLA-*DRA*, *CD46*, *FCER1G*) (**Figures 4F** and **S3A**). Finally, expression of genes in cluster 4 (325) significantly decreased 3 DPC in the day -14 group, 6 and 9 DPC in the controls, and 9 DPC in the day -7 group. Those genes enriched to GO terms associated to “lymphocyte activation” (*CD3D*, *CD27*, *IGHD*, *IGLC1*, *CSK*, *TNFRSF1B*) and “antigen processing and presentation” (*HLA-B*, *HLA-C*, *HLA-DMB*, *HLA-E*, *CTSB*) (**Figures 4F** and **S3A**).

### Signature Transcriptional Profiles Are Associated With Differential Disease Outcomes in Animals Vaccinated 3 Days Before Challenge

**Figure 5:**
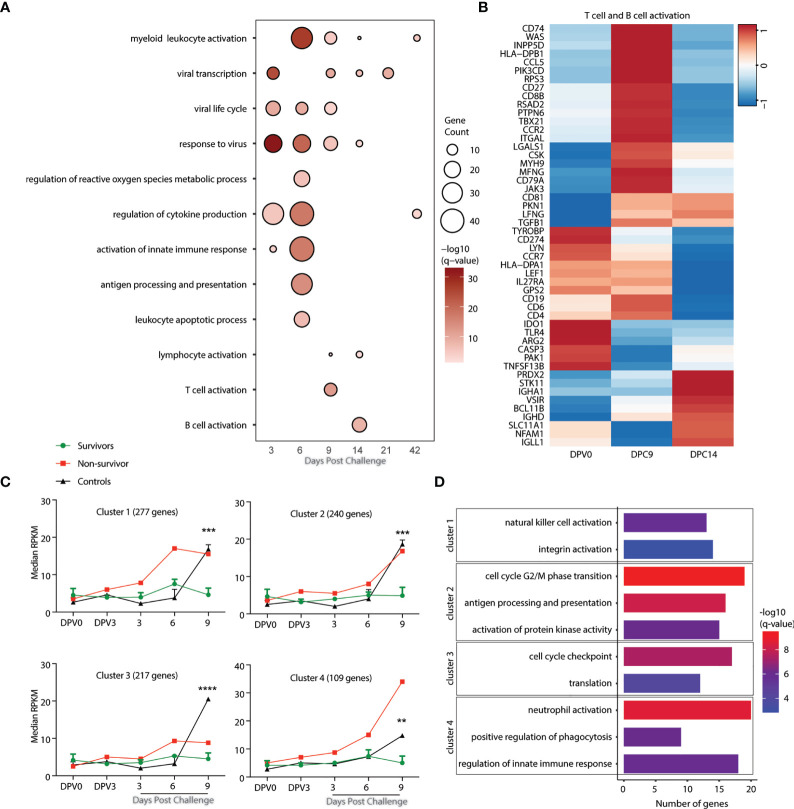
Gene signatures of fatality versus survival in animals vaccinated 3 days before challenge. **(A)** Functional enrichment of DEGs post VSV-MARV challenge day -3 group; each bubble represents a Gene Ontology (GO) term, and the size of each bubble represents number of DEGs enriching to that GO terms while color intensity represents the statistical significance (shown as –log_10_ of the q-corrected p-value). **(B)** Heatmap of DEGs detected post challenge in the day -3 group enriching to “T cell activation”, and “B cell activation.” Each column represents the median normalized transcript counts (RPKM) for each gene at each time point. The range of colors is based on scaled and centered RPKM values of the entire set of genes, with red indicating highly expressed genes and blue indicating lowly expressed genes. **(C)** maSigPro analysis identified four distinct and significant temporal expression clusters of genes between survivor and non-survivor animals. The median RPKM of each cluster over time is plotted. Statistical significance among time points was determined using one-way ANOVA with Dunnett’s multiple-comparison test relative to 0 DPV. Statistical significance is indicated as ****p < 0.0001, ***p < 0.001, and **p < 0.01. **(D)** Functional enrichment of the four clusters presented in panel (C). Horizontal bars represent the number of genes mapping to each GO term with color intensity representing the negative log of the FDR-adjusted p-value (-log(q-value).

VSV-MARV vaccination 3 days before challenge led to a mixed outcome with 3 animals surviving and 1 animal succumbing to MARV infection. We first carried out pairwise analysis of the transcriptional profiles of the samples in animals that survived challenge relative to 0 DPV. DEGs were detected at each DPC relative to 0 DPV peaking at 6 DPC (**Table 1**). DEGs that enriched to GO terms “viral transcription” (e.g., *POLR2E*, *POLR2F*, *RPL8*, *RPS27A*, and *RPS28*); “myeloid leukocyte activation” (*SERPINB1*, *MYD88*, *CD14*, *TLR4*, and *TLR8*); and several other immune related GO terms (*MX1*, *OAS1*, *OAS2*, *STAT2*, *S100A8*, *S100A12, STAT1, HERC5*, and *TGFB1*) were upregulated at 3 and 6 DPC (**Figures 5A** and **S3B, C**). Genes important for antigen processing and presentation, and T and B cell activation were detected 6, 9, and 14 DPC, respectively (**Figure 5A**). DEGs involved in “antigen processing and presentation” included MHC/HLA molecules (*HLA-B*, *HLA-C*, *HLA-DQB1*) and components of the proteosome (*PSMA3*) (**Figure S3C**). Genes encoding HLA molecules were mostly downregulated while genes encoding proteosome subunits were upregulated. DEGs mapping to “T cell activation” and “B cell activation” include *CD4*, *CD8B*, *CCL5*, *TNFSF13B*, *CD274*, *IL2RA*, *CD19*, *CD27*, *IGHA1*, and *IGHD* the expression of which peaked 9 DPC (**Figure 5B**).

To uncover gene expression signatures that distinguish survival versus fatal outcome, we analyzed transcriptional changes 0 DPV, 0, 3, 6, and 9 DPC using maSigPro with all NHPs vaccinated on day -3. Animals were divided into survivors (n=3), non-survivor (n=1), and controls (n=2) for the analysis. This approach identified 4 clusters of genes with temporal expression changes that were significantly different between the groups (**Figure 5C**). Genes in clusters 1 (277), 2 (240), and 4 (109) were upregulated in both the negative controls as well as the animal that succumbed to disease. Genes in cluster 1 play a role in natural killer cell activation (e.g. *VAMP7*, *SLAMF7*, and *SNX27*) and integrin activation (*RAP1B*, *ITGB1BP1*, and *P2RY12*) (**Figures 5C, D** and **S3D**). Genes in cluster 2 genes enriched to cell cycle G2/M phase transition (*PSMB5*, *PSMC1*, *KHDRBS1*, and *CCNB1*); antigen processing and presentation (*KLC1* and *DCTN2*); and signaling (*MAP3K20*, *KIDINS220*, *PARK7*) (**Figures 5C, D** and **S3D**). Cluster 4 genes mapped to GO terms including neutrophil activation (*PNP*, *CD63*, and *FCAR*); positive regulation of phagocytosis (*FCER1G*, *FPR2*, and *PTX3*); and regulation of innate immune response (*IRF7*, *TRIM21*, *IL18RAP*, and *SOCS3*) (**Figures 5C, D** and **S3D**). Genes in cluster 3 were significantly upregulated only in the negative control animals at 9 DPC. Those genes enriched to cell cycle checkpoints (*CDK1* and *CDK5RAP2*) and translation (*EIF2D* and *EIF4B*) (**Figures 5C, D** and **S3D**).

To further capture any transcriptional differences between the 3 surviving animals and the animal that succumbed to MARV challenge, we compared the WB transcriptional landscape between these animals at 0 DPV. This comparison identified 55 upregulated genes in the surviving relative to non-surviving animal on the day of vaccination some of which play a role in myeloid leukocyte activation (*CD14*, *GRN*, *HEXIM1*, and *SIGLEC5*); regulation of MAPK cascade (*INPP5K*, *CEACAM1*, and *MINK1*); and regulation of cell adhesion (*ST3GAL4*, *ADAM8*, and *LAMA5*) (**Figures S3E, F**).

## Discussion

In a previous study we demonstrated that a single dose of this VSV-based MARV vaccine administered 35 days prior to challenge is highly efficacious protecting 100% of vaccinated NHPs from MVD (8). Here, we determined the minimum time between vaccination and challenge with a single dose of 1x 10^7^ PFU. NHPs vaccinated 14 and 7 days prior to challenge with a lethal dose of MARV were uniformly protected from lethal MVD, while vaccination 3 days before challenge resulted in 75% survival. A previous study with VSV-MARV demonstrated the potential of this vaccine to be used as an effective post-exposure treatment. When VSV-MARV was administered 20-30 min after lethal MARV challenge, all treated NHPs survived the challenge without signs of MVD (9). This result highlighted the fast-acting potential of this vaccine which was investigated in detail here. In addition, our observations are very similar to a study conducted with the FDA-approved VSV-EBOV vaccine against EBOV in NHPs where 67% of animals survived when animals were vaccinated 3 days before challenge (12), indicating that this platform can provide rapid protection and is suitable for deployment during an outbreak as has been described for VSV-EBOV (13).

Protection against filovirus infection and disease using VSV-based vaccines is mainly mediated by antigen-specific humoral immune responses (15, 24). The dynamics of MARV GP-specific IgM and IgG responses demonstrated here were very similar to those reported for VSV-EBOV peaking around 10-14 days post vaccination (12). Although VSV-EBOV induced neutralizing IgM antibodies in humans (25), neutralizing antibody responses were not examined here as previous studies showed that they do not correlate with outcome after MARV infection (26). The development of the antibody response correlates with the induction of genes important for lymphocyte activation and function 11 and 14 DPV. Specifically, differential gene expression revealed a significant over-representation of genes that enrich to GO terms associated with B cell activation, humoral responses, T cell migration, and lymphocyte mediated immunity. Notable DEGs detected at these time points encoded heavy chains and factors important for memory B cell differentiation. These observations are in line with those recently reported for WB analysis following VSV-EBOV vaccination as well as PBMC analysis following VSV-MARV vaccination (8).

Since previous studies have shown limited contributions of T cells to vaccine-mediated protection against filoviruses (15, 27, 28), we did not measure the frequency of MARV GP-specific T cells in this study. Nevertheless, gene expression changes associated with T cell activation were evident 11-14 DPV. For instance, expression of genes important for TCR signaling and T cell survival were upregulated 11 DPV, and their expression remained increased at 14 DPV. Additionally, expression of genes important for antigen presentation, especially those encoding MHC molecules increased significantly 11 DPV. Overall, these findings support a role for T cells in mediating protection, most likely by providing help for affinity maturation and class switching of B cells as well as other potentially anti-viral functions as indicated by previous T cell depletion studies for VSV-EBOV (15, 29).

VSV-MARV vaccination also induced a tightly regulated innate immune response. Several ISGs, Dead Box helicases and nucleic acid sensors were acutely induced 3 DPV. Interestingly, this induction was less robust than that observed for VSV-EBOV as indicated by data presented here and published elsewhere (29–31). In contrast, antiviral innate immune responses in control animals and those that succumb to infection are progressively amplified as disease progresses and are associated with adverse outcomes and high mortality both in NHP and humans (3, 4, 16, 32–35). Although circulating levels of type I IFN do not significantly change with VSV-based vaccination, it is possible that the local production of IFNα/β in draining lymph nodes is sufficient to induce substantial expression of these ISGs (36). Moreover, control animals generated an excessive inflammatory response punctuated by substantial upregulation of genes encoding cytokines and chemokines and those important for neutrophil activation and function. This is in line with previous reports of neutrophilia and cytokine storm following lethal challenge with both EBOV and MARV (8, 12, 16, 27, 37–40). Finally, robust induction of genes that play a role in wound healing was also detected in control animals, indicative of the coagulopathy that accompanies EVD and MVD (37, 39, 40).

Innate antiviral responses have been shown to mediate the rapid protection using the VSV-EBOV vaccine in NHPs as determined by transcriptional analysis (28). Similarly, animals that were vaccinated 3 days before lethal challenge and survived exhibited hallmarks of robust innate immune responses that were attenuated by 14 DPC in line with resolution of the mild to moderate MVD symptoms they experienced. In contrast, the animal that succumbed to infection experienced sustained innate immune activation, and significant upregulation of genes that play a role in neutrophil activation. Interestingly, expression of genes important for myeloid cell activation and innate immunity was lower in blood samples obtained from this animal before vaccination. These observations, albeit preliminary, suggest that baseline expression of components of the innate immune branch may regulate individual responses to vaccination (and infection) and may modulate susceptibility to disease. This question merits additional investigation.

Another important observation in this study is that the robust innate immune responses induced by VSV-EBOV failed to protect animals from lethal MARV challenge when administered close to the time of challenge. In other words, the induction of ISGs by itself was not sufficient to “buy time” for antigen-specific responses to develop in animals vaccinated close to challenge. These findings suggest that MARV GP modulates the quality of the innate immune response and that antigen-specific responses induced by the vaccine rather than the virus under those circumstances are critical for shaping a protective immune response.

In summary, this study demonstrates that the VSV-MARV vaccine provides uniform protection within 7 days after administration of a single dose. In addition, 75% of the NHPs vaccinated only 3 days prior to challenge survived the lethal MARV infection. This rapid protection described here is comparable to that observed following administration of the approved VSV-EBOV vaccine (12) highlighting the fast-acting potential of this vaccine platform. VSV-EBOV was successfully used in a ring-vaccination approach in 2015 during the West African EBOV epidemic (13). In light of the first ever identified MARV case in Guinea in August 2021 (41) this study promotes the use of the VSV-MARV as an emergency vaccine suitable for ring-vaccination approaches.

## Data Availability Statement

The datasets presented in this study can be found in online repositories. The names of the repository/repositories and accession number(s) can be found below: NCBI SRA BioProject, accession no: PRJNA763231.

## Ethics Statement

The animal study was reviewed and approved by the Rocky Mountain Laboratories Animal Care and Use Committee, NIAID.

## Author Contributions

AM designed the study. AM and IM secured funding. AM, FF, and PH performed the animal studies. AM, AJ, ARM, JC, KO’D, and AP processed the samples and performed assays. AM, AJ, ARM, KO’D, AP, and IM analyzed the data. AM and IM wrote the manuscript. All authors contributed to the article and approved the submitted version.

## Funding

This work was supported by the Intramural Research Program NIAID, NIH and in part by the National Center for Research Resources and the National Center for Advancing Translational Sciences, NIH, through grant UL1 TR001414 awarded to IM.

## Conflict of Interest

The authors declare that the research was conducted in the absence of any commercial or financial relationships that could be construed as a potential conflict of interest.

## Publisher’s Note

All claims expressed in this article are solely those of the authors and do not necessarily represent those of their affiliated organizations, or those of the publisher, the editors and the reviewers. Any product that may be evaluated in this article, or claim that may be made by its manufacturer, is not guaranteed or endorsed by the publisher.

## Supplementary Material

Supplementary Figure 1 Blood cell counts and serum chemistry of NHPs.

Supplementary Figure 2 Cytokine response in vaccinated and challenged NHPs.

Supplementary Figure 3 Transcriptional changes after challenge.
