# Supplementary material for: Single Dose of a VSV-Based Vaccine Rapidly Protects Macaques From Marburg Virus Disease
Source: Front Immunol. 2021 Oct 27;12:774026. doi: 10.3389/fimmu.2021.774026 (PMC8578864; doi:10.3389/fimmu.2021.774026)
Supplement: Supplementary Figure 1 — Blood cell counts and serum chemistry of NHPs. [file Presentation_1.pdf]

Single dose of a VSV-based vaccine rapidly protects macaques from Marburg virus disease

Andrea Marzi[1], Allen Jankeel[2], Andrea R. Menicucci[2], Julie Callison[1], Kyle L. O'Donnell[1], Friederike Feldmann[3], Amanda N. Pinski[2], Patrick W. Hanley[3], Heinz Feldmann[1], and Ilhem Messaoudi[2]

[1]Laboratory of Virology, Division of Intramural Research, National Institute of Allergy and Infectious Diseases, National Institutes of Health, Hamilton, MT 59840, USA
[2]Department of Molecular Biology and Biochemistry, University of California, Irvine, Irvine, CA 92697, USA
[3]Rocky Mountain Veterinary Branch, Division of Intramural Research, National Institute of Allergy and Infectious Diseases, National Institutes of Health, Hamilton, MT 59840, USA

Supplemental Materials
Figure S1. Blood cell counts and serum chemistry of NHPs.
Figure S2. Cytokine response in vaccinated and challenged NHPs.
Figure S3. Transcriptional changes after challenge.

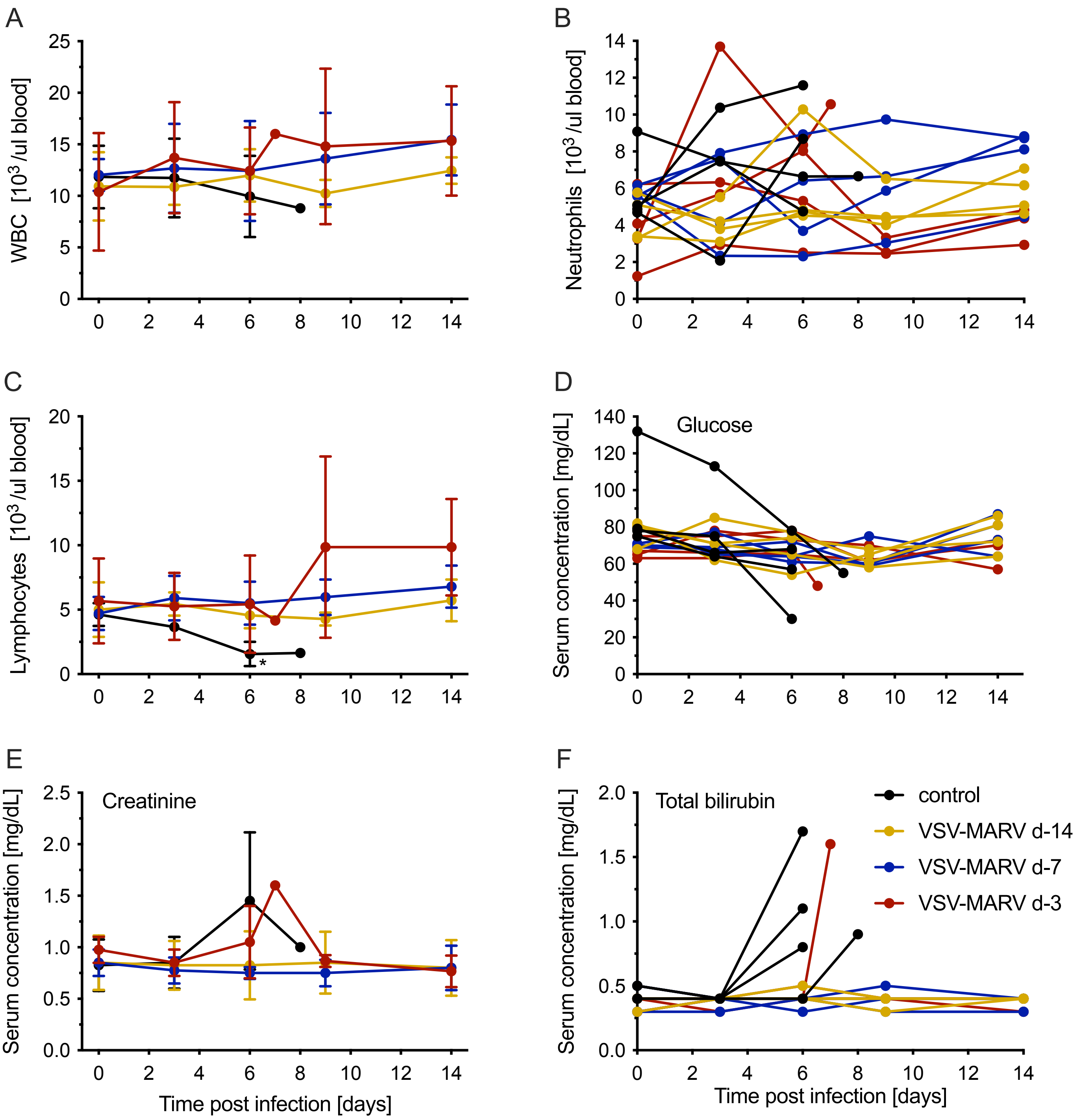

Figure S1. Blood cell counts and serum chemistry of NHPs. Levels of (A) white blood cells (WBC), (B) neutrophils, and (C) lymphocytes in whole blood samples over time are shown. Serum concentration of (D) glucose, (E) creatinine, and (F) total bilirubin over the course of the study are depicted. Mean and SD are depicted in (A, C, E). Statistical significance among groups was determined using two-way ANOVA with Tukey's multiple comparisons. Statistical significance is indicated as $^*p < 0.05$.

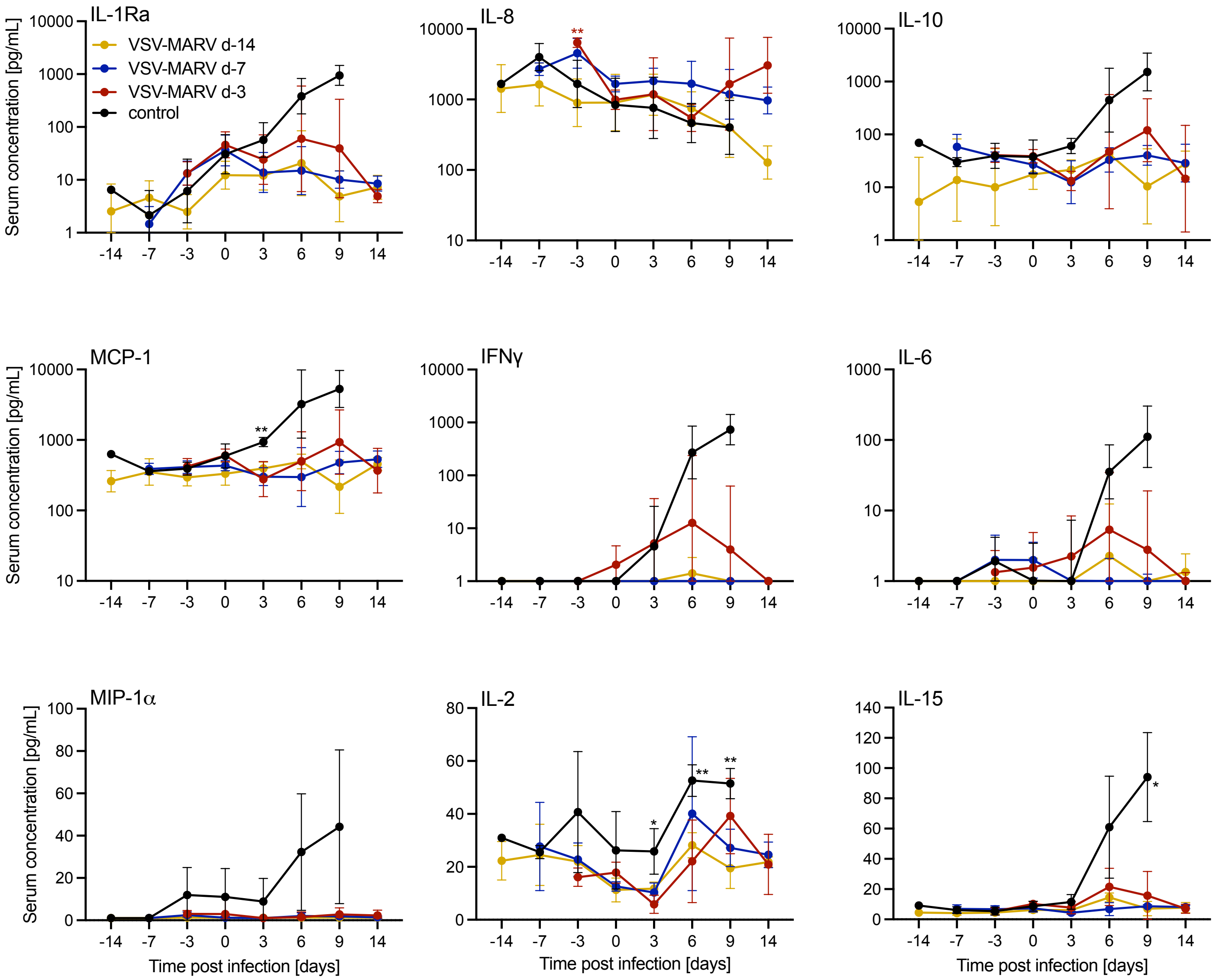

Figure S2. Cytokine response in vaccinated and challenged NHPs. The concentration of selected cytokines in the serum of vaccinated and challenged NHPs was determined over time. Geometric mean and geometric SD are shown for IL-1Ra, IL-8, IL-10, MCP-1, IFNg, and IL-6. Mean and SD are depicted for MIP-1a, IL-2 and IL-15. Statistical significance among groups was determined using two-way ANOVA with Tukey's multiple comparisons. Statistical significance is indicated as **p < 0.01, and *p < 0.05.

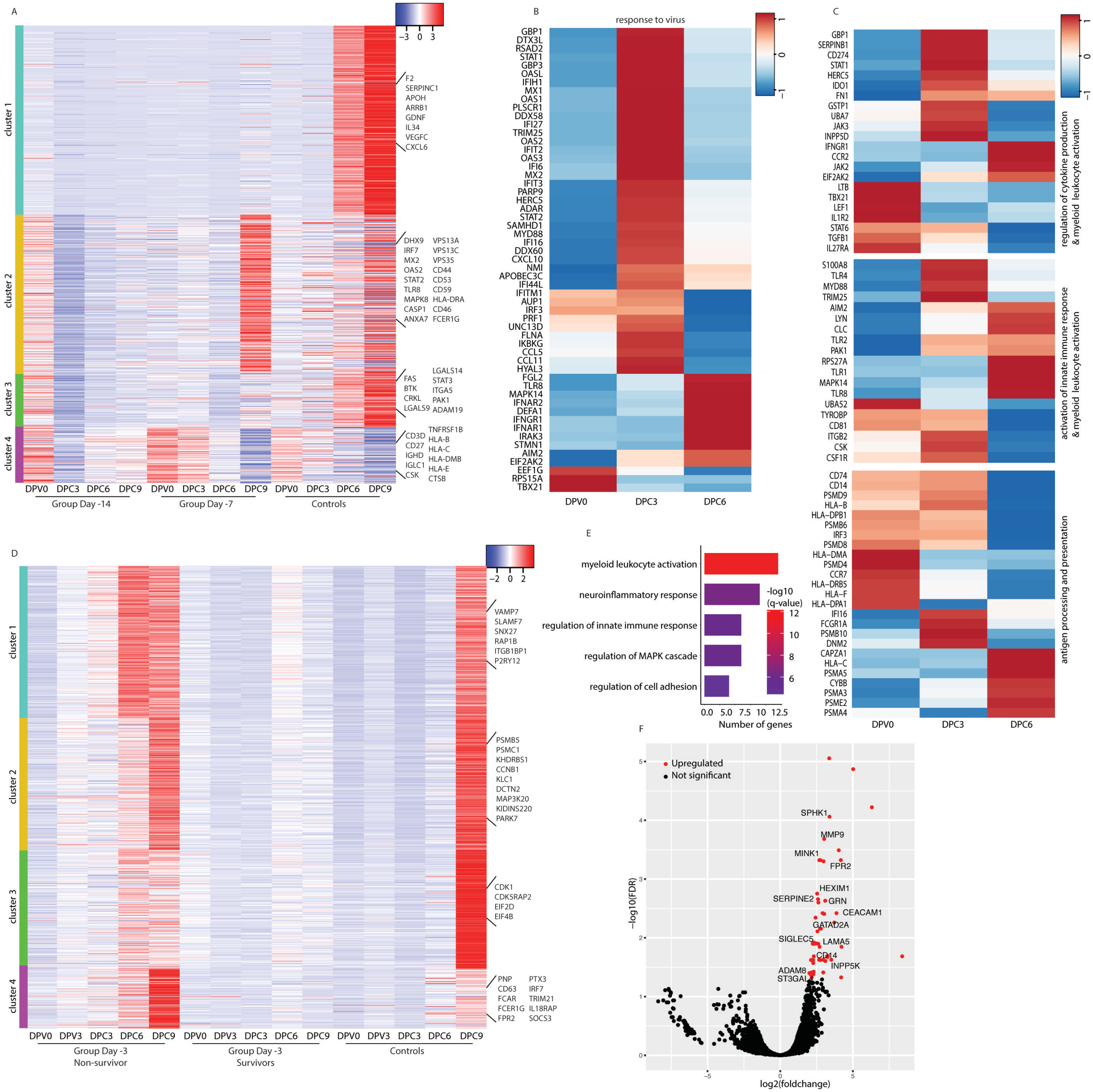
A
−3 0 3
cluster 1
cluster 2
cluster 3
cluster 4
F2
SERPINC1
APOH
ARRB1
GDNF
IL34
VEGFC
CXCL6
DHX9 VPS13A
IRF7 VPS13C
MX2 VPS35
OAS2 CD44
STAT2 CD53
TLR8 CD59
MAPK8 HLA-DRA
CASP1 CD46
ANXA7 FCER1G
FAS LGALS14
BTK STAT3
CRKL ITGA5
LGALS9 PAK1
ADAM19
CD3D TNFRSF1B
CD27 HLA-B
IGHD HLA-C
IGLC1 HLA-DMB
CSK HLA-E
CTSB
DPV0 DPC3 DPC6 DPC9 DPV0 DPC3 DPC6 DPC9 DPV0 DPC3 DPC6 DPC9
Group Day -14
Group Day -7
Controls
B
response to virus
1
0
−1
GBP1
DTX3L
RSAD2
STAT1
GBP3
OASL
IFIH1
MX1
OAS1
PLSCR1
DDX58
IFI27
TRIM25
OAS2
IFIT2
OAS3
IFI6
MX2
IFIT3
PARP9
HERC5
ADAR
STAT2
SAMHD1
MYD88
IFI16
DDX60
CXCL10
NMI
APOBEC3C
IFI44L
IFITM1
AUP1
IRF3
PRF1
UNC13D
FLNA
IKBKG
CCL5
CCL11
HYAL3
FGL2
TLR8
MAPK14
IFNAR2
DEFA1
IFNGR1
IFNAR1
IRAK3
STMN1
AIM2
EIF2AK2
EEF1G
RPS15A
TBX21
DPV0 DPC3 DPC6
C
1
0
−1
GBP1
SERPINB1
CD274
STAT1
HERC5
IDO1
FN1
GSTP1
UBA7
JAK3
INPP5D
IFNGR1
CCR2
JAK2
EIF2AK2
LTB
TBX21
LEF1
IL1R2
STAT6
TGFB1
IL27RA
regulation of cytokine production & myeloid leukocyte activation
S100A8
TLR4
MYD88
TRIM25
AIM2
LYN
CLC
TLR2
PAK1
RPS27A
TLR1
MAPK14
TLR8
UBA52
TYROBP
CD81
ITGB2
CSK
CSF1R
activation of innate immune response & myeloid leukocyte activation
CD74
CD14
PSMD9
HLA−B
HLA−DPB1
PSMB6
IRF3
PSMD8
HLA−DMA
PSMD4
CCR7
HLA−DRB5
HLA−F
HLA−DPA1
IFI16
FCGR1A
PSMB10
DNM2
CAPZA1
HLA−C
PSMA5
CYBB
PSMA3
PSME2
PSMA4
antigen processing and presentation
DPV0 DPC3 DPC6
D
−2 0 2
cluster 1
cluster 2
cluster 3
cluster 4
VAMP7
SLAMF7
SNX27
RAP1B
ITGB1BP1
P2RY12
PSMB5
PSMC1
KHDRBS1
CCNB1
KLC1
DCTN2
MAP3K20
KIDINS220
PARK7
CDK1
CDK5RAP2
EIF2D
EIF4B
PNP PTX3
CD63 IRF7
FCAR TRIM21
FCER1G IL18RAP
FPR2 SOCS3
DPV0 DPV3 DPC3 DPC6 DPC9 DPV0 DPV3 DPC3 DPC6 DPC9 DPV0 DPV3 DPC3 DPC6 DPC9
Group Day -3 Non-survivor
Group Day -3 Survivors
Controls
E
myeloid leukocyte activation
neuroinflammatory response
regulation of innate immune response
regulation of MAPK cascade
regulation of cell adhesion
-log10 (q-value)
12
10
8
6
0.0 5.0 7.5 10 12.5
Number of genes
F
Upregulated
Not significant
SPHK1
MMP9
MINK1
FPR2
HEXIM1
SERPINE2
GRN
CEACAM1
GATAD2A
SIGLEC5
LAMA5
CD14
INPP5K
ADAM8
ST3GAL4
−log10(FDR)
0 1 2 3 4 5
−5 0 5
log2(foldchange)

Figure S3. Transcriptional changes after challenge. (A, D) Heatmaps representing four clusters of genes significantly associated with clinical outcome identified by maSigPro in **(A)** the day -14, day -7 vaccine groups and controls or **(D)** in the day -3 vaccine group and controls. Each column represents the median normalized transcript counts (RPKM) for each gene at each time point. The range of colors is based on scaled and centered RPKM values of the entire set of genes, with red indicating highly expressed genes and blue indicating lowly expressed genes. **(B, C)** Heatmaps of DEGs detected post VSV-MARV challenge in the day -3 vaccine group enriching to "response to virus", and "regulation of cytokine production", "activation of innate immune response", "myeloid leukocyte activation", and "antigen processing and presentation." Each column represents the median normalized transcript counts (RPKM) for each gene at each time point. The range of colors is based on scaled and centered RPKM values of the entire set of genes, with red indicating highly expressed genes and blue indicating lowly expressed genes. **(E)** Volcano plot depicting DEGs detected 0 DPV between animals that survived versus the animal that succumbed to MARV challenge in the day-3 vaccine group. **(F)** Functional enrichment of the 55 DEGs detected in panel E. Horizontal bars represent the number of genes mapping to each GO term with color intensity representing the negative log of the FDR-adjusted p-value -log(q-value).
